# Supplementary material for: Heat adaptation of phage T7 under an extended genetic code
Source: Virus Evol. 2021 Dec 1;7(2):veab100. doi: 10.1093/ve/veab100 (PMC8923235; doi:10.1093/ve/veab100)
Supplement: veab100_Supp [file veab100_supp.zip › Sequencing_Analysis.zip!/Sequencing_Analysis/A2/evidence/MC_SIDE_1_42.html]

BRESEQ :: Evidence


| Missing coverage evidence... | | | | | | | | | | |
| --- | --- | --- | --- | --- | --- | --- | --- | --- | --- | --- |
|  |  |  | seq id | start | end | size | ←reads | reads→ | gene | description |
| \* | \* | ÷ | NC\_001604.1 | 39743 | 39937 | 195 | 241 [230] | [0] NA | *–/–* | –/– |

Only 100 of 241 total aligned reads displayed.  

`TGTGGTACTTTGTGCTGCCCTTGGGTACGCATCTCTTACTGGAGACCTCAGTTCACTGGAGTCTGTCGTTTGCTCTATACTCACTTGTAGCGATTAGGGTCTTCCTGACCGACTGATGGCTCACCGAGGGATTCAGCGGTATGATTGCATCACACCACTTCATCCCTATAGAGTCAAGTCCTAAGGTATACCCATAAAGAGCCTCTAATGGTCTATCCTAAGGTCTATACCTAAAGATAGGCCATCCTATCAGTGTCACCTAAAGAGGGTCTTAGAGAGGGCCTATGGAGTTCCTATAGGGTCCTTTAAAATATACCATAAAAATCTGAGTGACTATCTCACAGTGTACGGACCTAAAGTTCCCCCATA‑GGGGGTACCTAAAGCCCAGCCAATCACCTAAAGTCAACCTTCGGTTGACCTTGAGGGTTCCCTAAGGGTTGGGGATGACCCTTGGGTTTGTCTTTGGGTGTTACCTTGAGTGTCTCTCTGTG‑‑‑‑‑‑‑‑‑‑‑‑‑‑‑‑‑‑‑TCCCT  >  NC_001604.1/39442‑39937`  

                                                                                                                                                                                                                                                                                                            `|`                                                                                                                                                                                                                         
`tgtgGTACTTTGTGCTGCCCGTGGGTACGCATCTCTTACTGGAGACATCAGTTCACTGGAGTCTGTCGTGTGCTCTATACTCACTTGTAGCGATTAGGGTCTGCCTGACCGACTGATGGCTCACCGAGGGATTCAGCGGTATGATTGCATCACACCACTTCATCCCTATAGAGTCAAGTCCTAAGGTATACCCATAAAGAGCCTCTAATGGTCTATCCTAAGGTCTATACCTAAAGATAGGCCATCCTATCAGTGTCACCTAAAGAGGGTCTTAGAGAGGGCCTATGGAGTTCCTATAggg                                                                                                                                                                                                                         <  1:659799/301‑1 (MQ=255)`  
`tgtgGGACTTTGTGCTGCTCTTGGGTACGCATCTCTTACTGGAGACCTCAGTTCACTGGAGTCTGTCGTTTGCTTTATACTCACTTGTAGCGATTAGGGTCTTCCTGACCGACTGATGGCTCACCGAGGGATTCAGCGGTATGATTGCATCACACCACTTCATCCCTATAGAGTCAAGTCCTAAGGTATACCCATAAAGAGCCTCTAATGGTCTATCCTAAGGTCTATACCTAAAGATAGGCCATCCTATCAGTGTCACCTAAAGAGGGTCTTAGAGAGGGCCTATGGAGTTCCTATAggg                                                                                                                                                                                                                         <  1:372804/301‑1 (MQ=255)`  
`gtgggctcggagatgtgtataagagacagCATCTCTTACTGGAGACCTCAGTTCACTGGAGTCTGTCGTTTGCTCTATACTCACTTGTAGCGATTAGGGTCTTCCTGACCGACTGATGGCTCACCGAGGGATTCAGCGGTATGATTGCATCACAACACTTCATCCCTATAGAGTCAAGTCCTAAGGTATACCCATAAAGAGCCTCTAATGGTCTATCCTAAGGTCTATACCTAAAGATAGGCCATCCTATCAGTGTCACCTAAAGAGGGTCTTAGAGAGGGCCTATGGAGTTCCTATAggg                                                                                                                                                                                                                         <  1:577776/273‑1 (MQ=255)`  
 `ttgGTACTTTGGGCTGCCCTTGGGTACGCAGCTCTTACTGGAGACCTAAGTTCACTGGAGTCTGTCGTTTGCTCTATACTCACTTGTAGCGATTAGGGTCTTCCGGACAGACTGATGGCTCACCGAGGGATTCAGCGGTATGATTGCATCACACCACTTCATCCATATAGAGGCAAGTCCTAAGGTATACCCATAAAGAGCATCTAATGGTCTATCCTAAGGTCTATACCTAAAGATAGGCCATCCTATCAGTGTCACCTAAAGAGGGTCTTAGAGAGGGCCTATGGAGTTCCTATAGGGt                                                                                                                                                                                                                        <  1:206836/300‑1 (MQ=255)`  
   `ggTACTTTGTGCTGCCCTTGGGTACGCATCTCTTACTGGAGACCTCAGTTCACTGGAGTCTGTCGTTTGCTCTATACTCACTTGTAGCGATTAGGGTCTTCCTGACCGACTGATGGCTCACCGAGGGATTCAGCGGTATGATTGCATCACACCACTTCATCCCTATAGAGTCAAGTCCTAAGGTATACCCATAAAGAGCCTCTAATGGTCTATCCTAAGGTCTATACCTAAAGATAGGCCATCCTATCAGTGTCACCTAAAGAGGGTCTTAGAGAGGGCCTATGGAGTTCCTATAGGGTcc                                                                                                                                                                                                                      <  1:734345/301‑1 (MQ=255)`  
      `aCTTTGTGCTGCCCTTGGGTACGCATCTCTTACTGGAGACCTCAGTTCACTGGAGTCTGTCGTTTGCTCTATACTCACTTGTAGCGATTAGGGTCTTCCTGACTGACTGATGGCTCACCGAGGGATTCAGCGGCATGATTGCATCACACCACTTCATCCCTATAGAGTCAAGTCCTAAGGTATACCCATAAAGAGCCTCTAATGGTCTATCCTAAGGTCTATACCTAAAGATAGGCCATCCTATCAGTGTCACCTAAAGAGGGTCTTAGAGAGGGCCTATGGAGTTCCTATAGGGTCCttt                                                                                                                                                                                                                   >  1:409452/1‑301 (MQ=255)`  
          `ccggagatgtgtataagagacagtctTACTGGAGACCTCAGTTCACTGGAGTCTGTCGTTTACTCTATACTCACTTGTAGCGATTAGGGTCTTACGGAACGACTGATGGCTCACCGAGGGATTCAGCGGTATGATTGCAACACAGAACTTCATCCCTATAGAGTCAAGTACTAAGGTATACCCATAAAGAGCATCTAATGGTATATCCTAAGGTCTATACCTAAAGATAGGCCATCCTATCAGTGTAACCTAAAGAGGGTCTTAGAGAGGGCCTATGGAGTTCCTATAGGGTCCTTTaaaa                                                                                                                                                                                                               <  1:727607/278‑1 (MQ=255)`  
           `gtgCTGCCCTTGGGTACGCATCTCTTACTGGAGACCTCAGTTCACTGGAGTCTGTCGTTTGCTCTATACTCACTTGTAGCGATTAGGGTCTTCCTGACCGACTGATGGCTCACCGAGGGATTCAGCGGTATGATTGCATCACACCACTTCATCCCTATAGAGTCAAGTCCTAAGGTATACCCATAAAGAGCCTCTAATGGTCTATCCTAAGGTCTATACCTAAAGATAGGCCATCCTATCAGTGTCACCTAAAGAGGGTCTTAGAGAGGGCCTATGGAGTTCCTATAGGGTCCTTTAAAat                                                                                                                                                                                                              <  1:356666/301‑1 (MQ=255)`  
               `agagacagggTACGCATCTCTTACTGGAGACCTCAGTTCACTGGAGTCTGTCGTTTGCTCTATACTCACTTGTAGCGATTAGGGTCTTCCTGACAGACTGATGGCTCACCGAGGGATTCAGCGGTATGATTGCATCACACCACTTCATCCCTATAGAGTCAAGTCCTAAGGTATACCCATAAAGAGCCTCTAATGGTCTATCCTAAGGTCTATACCTAAAGATAGGCAATCCTATCAGTGTCACCTAAAGAGGGTCTTAGAGAGGGCCTATGGAGTTCCTATAGGGTCCTTTAAAATATAc                                                                                                                                                                                                          <  1:709066/294‑1 (MQ=255)`  
                      `gggTACGCATCTCTTACTGGAGACCTCAGTTCACTGGAGTCTGTCGTTTGCTCTATACTCACTTGTAGCGATTAGGATCTTCCTGACCGACTGATGGCTCACCGAGGGATTCAGCGGTATGATTGCATCACACCACTTCATCCCTATAGAGTCAAGTCCTAAGGTATACCCATAAAGAGCCTCTAATGGTCTATCCTAAGGTCTATACCTAAAGATAGGCCATCCTATCAGTGTCACCTAAAGAGGGTCTTAGAGAGGGCCTATGGAGTTCCTATAGGGTCCTTTAAAATATACCATaaaa                                                                                                                                                                                                   >  1:336889/1‑301 (MQ=255)`  
                       `ggTACGCATATCTTACTGGAGACCTCAGTTCACTGGAGTCTGTCGTTTGCTCTATACTCACTTGTAGCGATTAGGGTATTCCTGACCGACTGATGGCTCACCGAGGGATTCAGCGGTATGATTGCATCACACAACTTCATCCCTATAGAGTCAAGTCCTAAGGTATACACATAAAGAGCCTCTAATGGTCTATCCTAAGGTCTATACCTAAAGATAGGCCATCCTATCAGTGTCACCTAAAGAGGGTCTTAGAGAGGGCCTATGGAGTTCCTATAGGGTACTTTAAAATATACCATaaaaa                                                                                                                                                                                                  <  1:209786/301‑1 (MQ=255)`  
                             `tcggagatgtgtataagagacagtCACTGGAGTCTGTCGTTTGCTCTATACTCACTTGTAGCGATTAGGGTCTTCCTGACCGACTGATGGCTCACCGAGGGATTCATCGGTATGATTGCATCACACCACTTCATCCCTATAGAGTCAAGTCCTAAGGTATACCCATAAAGAGCCTCTAATGGTCTATCCTAAGGTCTATACCTAAAGATAGGCCATCCTATCAGTGTCACCTAAAGATGGTCTTAGAGAGGGCCTATGGAGTTCCTATAGGGTCCTTTAAAATATACCATAAAAATCTGAg                                                                                                                                                                                            <  1:560252/278‑1 (MQ=255)`  
                               `tctcTTACTGGAGACCTCAGTTCACTGGAGTCTGTCGTTTGCTCTATACTCACTTGTAGCGATTAGGGTCTTCCTGACCGACTGATGGCTCACCGAGGGATTCAGCGGTATGATTGCATCACACCACTTCATCCCTATAGAGTCAAGTCCTAAGGTATACCCATAAAGAGCCTCTAATGGTCTATCCTAAGGTCTATACCTAAAGATAGGCCATCCTATCAGTGTCACCTAAAGAGGGTCTTAGAGAGGGCCTATGGAGTTCCTATAGGGTCCTTTAAAatctgtctcttatacacatctc                                                                                                                                                                                          >  1:665097/1‑281 (MQ=255)`  
                                  `ctTACTGGAGACCTCAGTTCACTGGAGTCTGTCGTTTGCTCTATACTCACTTGTAGCGATTAGGGTCTTCCTGACCGACTGATGGCTCACCGAGGGATTCAGCGGTATGATTGCATCACACCACTTCATCCCTATAGAGTCAAGTCCTAAGGTATACCCATAAAGAGCCTCTAATGGTCTATCCTAAGGTCTATACCTAAAGATAGGCCATCCTATCAGTGTCACCTAAAGAGGGTCTTAGAGAGGGCCTATGGAGTTCCTATAGGGTCCTTTAAAATATAcctgtctcttatacacatct                                                                                                                                                                                       >  1:777066/1‑283 (MQ=255)`  
                                  `ctTACTGGAGACCTCAGTTCACTGGAGTCTGTCGTTTGCTCTATACTCACTTGTAGCGATTAGGGTCTTCATGACCGACTGATGGCTCACCGAGGGATTCAGCGGTATGATTGCATCACACCACTTCATCCCTATAGAGTCAAGTCCTAAGGTATACCCATAAAGAGCCTCTAATGGTCTATCCTAAGGTCTATACCTAAAGATAGGCCATCCTATCAGTGTCACCTAAAGAGGGTCTTAGAGAGGGCCTATGGAGTTCCTATAGGGTCCTTTAAAATATACCATAAAAATCTGAGTGACt                                                                                                                                                                                       >  1:679384/1‑301 (MQ=255)`  
                                         `gataCCTCAGTTCACTGGAGGCTGGCGTTTGCTCGATACTCACTAGTAGCGATTAGGGTCTTCCTGACCGACGGATGGCTCACCGATGGATTCAGCGGTATGATTGCATCACACCACGTCATCCCTATAGAGTCAAGTCCTAAGGTATACCCATAAAGAGCCTCTAATGGTCTATCCTAAGGTCTATACCTAAAGATAGGCCATCCTATCAGTGTCACCTAAAGAGGGTCTTAGAGAGGGCCTATGGAGTTCCTATAGGGTCCTTTAAAATATACCATAAAAATCTGAGTGACTATCTcac                                                                                                                                                                                <  1:153457/298‑1 (MQ=255)`  
                                         `gagaCCTCAGTTCACTGGAGTCTGTCGTTTGCTCTATACTCACTTGTAGCGATTAGGGTCTTCCTGACCGACTGATGGCTCACCGAGGGATTCAGCGGTATGATTGCATCACACCACTTCATCCCTATAGAGTCAAGTCCTAAGGTATACCCATAAAGAGCCTCTAATGGTCTATCCTAAGGTCTATACCTAAAGATAGGCCATCCTATCAGTGTCACCTAAAGAGGGTCTTAGAGAGGGCCTATGGAGTTCCTATAGGGTCCTTTAAAATATACCATAAAAATCTGAGTGACTATCTcac                                                                                                                                                                                <  1:359816/301‑1 (MQ=255)`  
                                         `gagaCCTCAGTTCACTGGAGTCTGTCGTTTGCTCTATACTCACTTGTAGCGATTAGGGTCTTCCTGACCGACTGATGGCTCACCGAGGGATTCAGCGGTATGATTGCATCACACCACTTCATCCCTATAGAGTCAAGTCCTAAGGTATACCCATAAAGAGCCTCTAATGGTCTATCCTAAGGTCTATACCTAAAGATAGGCCATCCTATCAGTGTCACCTAAAGAGGGTCTTAGAGAGGGCCTATGGAGTTCCTATAGGGTCCTTTAAAATATACCATAAAAATCTGAGTGACTATCTcac                                                                                                                                                                                <  1:684305/301‑1 (MQ=255)`  
                                           `tgggctcggagatgtgtataagagacaggCTCTATACTCACTTGTAGCGATTAGGGTCTTCCTGACCGACTGATGGCTCACAGAGGGATTCAGCGGTATGATTGCATCACACCACTTCATCACTATAGAGTCAAGTCCTAAGGTATACCAATAAAGAGCCTCTAATGGTCTATCCTAAGGTCTATACCTAAAGATAGGCCATCCTATCAGTGTCACCTAAAGAGGGTCTTAGAGAGGGCCTATGGAGTTCCTATAGGGTCCTTTAAAATATACCATAAAAATCTGAGTGAATATCTCACAg                                                                                                                                                                              <  1:81121/273‑1 (MQ=255)`  
                                           `tgggctcggagatgtgtataagagacaggCTCTATACTCACTTGTAGCGATTAGGGTATTCCTGACCGACTGATGGCTCACCGAGGGATTCAGCGGTATGATTGCATCACACCACTTCATCCCTATAGAGTCAAGTCCTAAGGTATACCCATAAAGAGCCTCTAATGGTCTATACTAAGGTCTATACCTAAAGATAGGCCATCCTATCAGTGTCACCTAAAGAGGGTCTTAGAGAGGGCCTATGGAGTTCCTATAGGGTCCTTTAAAATATACCATAAAAATCTGAGTGACTATCTCACAg                                                                                                                                                                              <  1:310548/273‑1 (MQ=255)`  
                                           `gaCCTCAGTTCACTGTAGTCTGTCGTTTGCTCTATACTCACTTGTAGCGATTAGTGTCTTCCTGACCGACTGATGGCTCACCGAGGGATTCAGCGGTATGATTGCATCACACCACTTCATCCCTATAGAGTCAAGTCCTAAGGTATACCCATAAAGAGCCTCTAATGGTCTATCCTAAGGTCTATACCTAAAGATAGGCCATCCTATCAGTGTCACCTAAAGAGGGTCTTAGAGAGGGCCTATGGAGTTCCTATAGGGTCCTTTAAAATATACCATAAAAATCTGAGTGACTATCTCACAg                                                                                                                                                                              <  1:765334/301‑1 (MQ=255)`  
                                           `gaCCTCAGTTCACTGGAGTCTGTCGTTTGCTCTATACTCACTTGTAGCGATTAGGGTCTTCCTGACCGACTGATGGCTCACCGAGGGATTCAGCGGTATGATTGCATCACACCACTTCATCCCTATAGAGTCAAGTCCTAAGGTATACCCATAAAGAGCCTCTAATGGTCTATCCTAAGGTCTATACCTAAAGATAGGCCATCCTATCAGTGTCACCTAAAGAGGGTCTTAGAGAGGGCCTATGGAGTTCCTATAGGGTCCTTTAAAATATACCATAAAAATCTGAGTGACTATCTCACAg                                                                                                                                                                              <  1:562962/301‑1 (MQ=255)`  
                                             `ccTCAGTTCACTGGAGTCTGTCGTTTGCTCTATACTCACTTGTAGCGATTAGGGTCTTCCTGACCGACTGATGGCTCACTGAGGGATTCAGCGGTATGATTGCATCACACCACTTCATCCCTATAGAGTCAAGTCCTAAGGTATACCCATAAAGAGCCTCTAATGGTCTATCCTAAGGTCTATACCTAAAGATAGGCCATCCTATCAGTGTCACCTAAAGAGGGTCTTAGAGAGGGCCTATGGAGTTCCTATAGGGTCCTTTAAAATATACCATAAAAATCTGAGTGAcctgtctcttata                                                                                                                                                                            >  1:343658/1‑289 (MQ=255)`  
                                                  `gTTCACTGGAGTCTGTCGTTTGCTCTATACTCACTTGTAGCGATTAGGGTCTTCCTGACCGACTGATGGCTCACCGAGGGATTCAGCGGTATGATTGCATCACACCACTTCATCCCTATAGAGTCAAGTCCTAAGGTATACCCATAAAGAGCCTCTAATGGTCTATCCTAAGGTCTATACCTAAAGATAGGCCATCCTATCAGTGTCACCTAAAGGGGGTCTTAGAGAGGGCCTATGTAGTTCCTATAGGGTCCTTTAAAATATACCATAAAAATCTGAGTGACTATCTCACAGTGTACgg                                                                                                                                                                       >  1:178403/1‑301 (MQ=255)`  
                                                   `aagatacagtgcTGGCGTTTGCTCCAAACTCACTTGTAGCGATTAGGGTCTTCCTGACCGACTGATGGCTCACCGATGGATTCAGCGGTATGATTGCATCACACCACTTCAGCCCTATAGAGTCAAGTCCTAAGGTATACCCATAAAGAGCCTCTAATGGTCTATCCTAAGGTCTATACCTAAATATAGGCCATCCTATCAGTGTCACCTAAAGAGGGTCTTAGAGAGGGCCTATGGAGTTCCTATAGGGTCCTTTAAAATATACCATAAAAATCTGAGTGACTATCTCACAGTGTACGGa                                                                                                                                                                      <  1:492271/290‑1 (MQ=255)`  
                                                    `tCACTGGAGTCTGTCGTTTGCTCTATACTCACTTGTAGCGATTAGGGTCTTCCTGACCGACTGATGGCTCACCGAGGGATTCAGCGGTATGATTGCATCACACCACTTCATCCCTATAGAGTCAAGTCCTAAGGTATACCCATAAAGAGCCTCTAATGGTCTATCCTAAGGTCTATACCTAAAGATAGGCCATCCTATCAGTGTCACCTAAAGAGGGTCTTAGAGAGGGCCTATGGAGTTCCTATAGGGTCCTTTAAAATATACCATAAAAATCTGAGTgctgtctcttatacacatctcc                                                                                                                                                                     >  1:221574/1‑280 (MQ=255)`  
                                                      `aCTGGAGTCTGTCGTTTGCTCTATACTCACTTGTAGCGATTAGGGTCTTCCTGAACGACTGATGGCTCACCGAGGGATTCAGCGGTATGATTGCATCACACCACTTCATCCCTATAGAGTCAAGTCCTAAGGTATACCCATAAAGAGCCTCTAATGGTATATCCTAAGGTCTATACCTAAAGATAGGCCATCCTATCAGTGTCACCTAAAGAGGGTCTTAGAGAGGGCCTATGGAGTTCCTATAGGGTCCTTTAAAATATACCATAAAAATCTGAGTGACTATCTCACAGTGTACGGAcat                                                                                                                                                                   >  1:380138/1‑299 (MQ=255)`  
                                                         `ggcgTCTGTCGTTTGCTCTATACTCACTTGTAGCGATTAGGGTCTTCCTGACCGACTGATGGCTCACCGAGTGATTCAGCGGTATGATTGCATCACACCACTTCATCCCTATAGAGTCAAGTCCTAAGGTATACCCATAAAGAGCCTCTAATGGTCTATCCTAAGGTCTATACCTAAAGATAGGCCATCCTATCAGTGTCACCTAAAGAGGGTCTTAGAGAGGGCCTATGGAGTTCCTATAGGGTCCTTTAAAATATACCATAAAAATCTGAGTGACTATCTCACAGTGTACGGACCTaca                                                                                                                                                                >  1:69638/4‑299 (MQ=255)`  
                                                           `aGTCTGTCGTTTGCTCTATACTCACTTGTAGCGATTAGGGTCTTCCTGACCGACTGATGGCTCACCGAGGGATTCAGCGGTATGATTGCATCAAACCACTTCATCCCTATAGAGTCAAGTCCTAAGGTATACCCATAAAGAGCCTCTAATGGTCTATCCTAAGGTCTATACCTAAAGATAGGCCATCCTATCAGGGTCACCTAAAGAGGGTCTTAGAGAGGGCCTATGGAGTTCCTATAGGGGGCTTTAAAATATACCATAAAAATATGAGAGACTATATCATAGTGTAAGGACCGAAAGt                                                                                                                                                              >  1:445920/1‑301 (MQ=255)`  
                                                                `gTCGTTTGCTCTATACTCACTTGTAGCGATTAGGGTCTTCCTGACCGACTGATGGCTCACCGAGTGATTCAGCGGTATGATTGCATCACACCACTTCATCCCTATAGAGTCAAGTCCTAAGGTATACCCATAAAGAGCCTCTAATGGTCTATCCTAATGTCTATACCTAAAGATAGGCCATCCTATCAGTGTCACCTAAAGAGGGTCTTAGAGAGGGCCTATGGAGTTCCTATAGTGTCCTTTAAAATATACCATAAAAATCTGAGTGACTATCTCACAGTGTACGGACCTAAAGTTcccc                                                                                                                                                         >  1:325248/1‑301 (MQ=255)`  
                                                                `gTCGTTTGCTCTATACTCACTTGTAGCGATTAGGGTCTTCCTGACCGACTGATGGCTCACCGAGGGATTCAGCGGTATGATTGCATCACACCACTTCATCCCTATAGAGTCAAGTCCTAAGGTATACCCATAAAGAGCCTCTAATGGTCTATCCTAAGGTCTATACCTAAAGATAGGCCATCCTATCAGTGTCACCTAAAGATGGTCTTAGAGAGGGCCTATGGAGTTCCTATCGGGTCCTTTAAAATATACCATAAAAATCTGAGTGACTATCTCACAgtgtctgtctcttatacccatc                                                                                                                                                         >  1:392787/1‑283 (MQ=255)`  
                                                                 `tCGTTTGCTCTATACTCACTTGTAGCGATTAGGGTCTTCCTGACCGACTGATGGCTCACCGAGGGATGCAGCGGTATGTTTGCATCACACCACTTCATCCCTATAGAGTCAAGTCCTAAGGTATACCCATAAAGAGCCTCTAATGGTCGATCCTAAGGTCTATACCTAAAGATAGGCCATCCTATCAGTGTCACCTAAAGAGGGTCTTAGAGAGGGCCTATGGAGTTCCTATAGGGTCCTTTAAAATATACCATAAAAATCTGAGTGACTATCTCACAGTGTACGGACCTAAAGTTccccc                                                                                                                                                        <  1:814155/301‑1 (MQ=255)`  
                                                                  `cGTTTGCTCTATACTCACTTGTAGCGATTAGGGTCTTCCTGACCGACTGATGGCTCACCGAGGGATTCAGCGGTATGATTGCATCACACCACTTCATCCCTATAGAGTCAAGTCCTAAGGTATACCCATAAAGAGCCTCTAATGGTCTATCCTAATGTCTATACCTAAAGATAGGCCATCCTATCAGTGTCACCTAAAGAGGGTCTTAGAGAGGGCCTATGGAGTTCCTTTAGGGTCCTTTAAAATATACCATAAAAATCTGAGTGACTATCTcacctgtctcttctccacatctccgagc                                                                                                                                                       >  1:166048/1‑276 (MQ=255)`  
                                                                       `gCTCTATACTCACTTGTAGCGATTAGGGTCTTCCTGACCGACTGATGGCTCACCTAGGGATTCAGCGGTATGATTGCATCACACCACTTCATCCCTATAGAGTCAAGTCCTAAGGTATACCCATAAAGAGCCTCTAATGGTCTATCCTAATGTCTATACCTAAAGATAGGCCATCCTATCAGTGTCACCTAAAGAGGGTCTTAGAGAGGGCCTATTGAGTTCCTATAGGGTCCTTTAAAATATACCATAAAAATCTGAGTGACTATCTCACAGTGTACGGACCTAAAGTTCCCCCATA‑ggg                                                                                                                                                 <  1:410500/301‑1 (MQ=255)`  
                                                                       `gCTCTATACTCACTTGTAGCGATTAGGGTCTTCCTGACCGACTGATGGCTCACCGAGGGATTCAGCGGTATGATTGCATCACACCACTTTATCCCTATAGAGTCAAGTCCTAAGGTATACCCATAAAGAGCCTCTAATGGTCTATCCTAAGGTCTATACCTAAAGATAGGCCATCCTATCAGTGTCACCTAAAGAGGGTCTTATAGAGGGCCTATGGAGTTCCTATAGGGTCCTTTAAAATATACCATAAAAATCTGAGTGACTATCTCACAgctgtctcttatacacatctccgagccca                                                                                                                                                  >  1:383419/1‑273 (MQ=255)`  
                                                                       `gCTCTATACTAACTTGTAGCGATTAGGGTCTTCCTGACCGACTGATGGCTCACCGAGGGATTCAGCGGTATGATTGCATCACACCACTTCATCCCTATAGAGTCAAGTCCTAAGGTATACCCATAAAGAGCCTCTAATGGTCTATCCTAAGGTCTATACCTAAAGATAGGCCATCCTATCAGTGTAACCTAAAGAGGGTCTTAGAGAGGGCCTATGGAGTTCCTATAGGGTCCTTTAAAATATACCATAAAAATCTGAGTGACTATCTCACAGTGTACGGACCTAAAGTTCCCCAATA‑ggg                                                                                                                                                 <  1:81137/301‑1 (MQ=255)`  
                                                                            `ataCTCACTTGTAGCGATTAGGGTCTTCCTGACCGACTGATGGCTCACCGAGGGATTCAGCGGTATGATTTCATCACACCACTTCATCCCTATAGAGTCAAGTCCTAAGGTATACCCATAAAGAGCCTCTAATGTTCTATCCTAAGGTCTATACCTAAAGATAGGCCATCCTATCAGTGTCACCTAAAGAGGGTCTTAGAGAGGGCCTATGGAGTTCCTATAGGGTCCTTTAAAATATACCATAAAAATCTGAGTGACTATCTCACAGTGTACGGActgtctcttatacacatctccgagc                                                                                                                                             >  1:399210/1‑277 (MQ=255)`  
                                                                               `agcACTTGTAGCGAGTAGGGTCTTCCTGACCGACTGATGGCTCACCGAGGGATTCAGCGGTATGATTGCATCAAACCACTTCATCCCTATAGAGTCAAGTCCTAAGGTATAACCATAAAGAGCCTCTAATGGTCTATCCTAAGGTCTATACCTAAAGATAGGCCATCCTATCAGTGTCACCTAAAGAGGGTATTAGAGAGGGCCTATGGAGTTCCTATAGGGTCCTGTAAAATATACCATAAAAATCTGAGTGACTATCTCACAGTGTACGGACCTAAAGTTCCCCCATA‑GGGGGTACCTa                                                                                                                                         <  1:469273/299‑1 (MQ=255)`  
                                                                                `ccACTTTTAGCGATTAGGGTCGTCCTGACCGACTGATGGCTCACCGAGGGATTCAGCGGTATGATTGCATCACACCACTTCATCCCTATAGAGTCAAGTCCTAAGGTATACCCATAAAGAGCCTCTAATGGTCTATCCTAAGGTCTATACCTAAAGATAGGCCATCCTATCAGTGTCACCTAAAGAGGGTCTTAGAGAGGGCCTATGGAGTTCCTATAGGGTCCTTTAAAATATACCATAAAAATCTGAGTGACTATCTCACAGTGTATGGACCTAAAGTTCCCCCATA‑GGGGGTACCTaa                                                                                                                                        <  1:274022/300‑1 (MQ=255)`  
                                                                                 `cACTTGTAGCGATTAGGGTCTTCCTGACCGACTGATGGCTCACCGAGGGATTCAGCGGTATGATTGCATCACACCACTTCATCCCTATAGAGTCAAGTCCTAAGGTATACCCATAAAGAGCCTCTAATGGTCTATCCTAAGGTCTATACCTAAAGATAGGCCATCCTATCAGTGTCACCTAAAGAGGGTCTTAGAGAGGGCCTATGGAGTTCCTATAGTGTCCTTTAAAATATACCATAAAAATCTGAGTGACTATCTCACAGTGTACGGACCTAAAGTT‑CCCCATA‑ggggctgtcccttt                                                                                                                                      >  1:754576/1‑291 (MQ=255)`  
                                                                                   `cTTGTAGCGATTAGGGTCTTCCTGACCGACTGATGGCTCACCGAGGGATTCAGCGGTATGATTGCATCACACCACTTCATCCCTATAGAGTCAAGTCCTAAGGTATACCCATAAAGAGCCTCTAATGGTCTATCCTAAGGTCTATACCTAAAGATAGGCCATCCTATCAGTGTCCCCTAAAGAGGGTCTTAGAGAGGGCCTATGGAGTTCCTATAGGGTCATTTAAAATATACCATAAAAATCTGAGTGACTATCTCACAGTGTACGGACCTAAAGTTCCCCCCTA‑ggggctgtctcttat                                                                                                                                     >  1:432370/1‑290 (MQ=255)`  
                                                                                            `aTTAGGGTCTTCCTGACCGACTGATGGCTCACCGAGGGATTCAGCGGTATGATTGCATCACACCACTTCATCCCTATAGAGTCAAGTCCTAAGGTATACCCATAAAGAGCCTCTAATGGTCTATCCTAATGTCTATACCTAAAGATAGGCCATCCTATCAGTGTCACCTAAAGAGGGTCTTAGAGAGGGCCTATGGAGTTCCTATAGGGTCCTTTAAAATATACCATAAAAATCTGAGTGACTATCTCACAGTGTACGGACCTAAAGTTcccccctgtctcttatacacatctccgagccc                                                                                                                             >  1:372495/1‑274 (MQ=255)`  
                                                                                                `gggTCTTCCAGACCGAATGATGGCTCACCGAGGGATTCAGCGGTATGATTGCATCACACCACTTCATACCTATAGAGTCAAGTCCTAAGGTATACCCATAAAGAGCCTCTAATGGGCTATCCTAAGGTCTATACCTAAAGATAGGCCATCCTATCAGTGTCACCTAAAGAGGGTCTTAGAGAGGGCCTATGGAGTTCCTATAGGGTCCTTTAAAATATACCATAAAAATCTGAGTGACTATCTCACAGTGTACGGACCTAAAGTTCCCCCATA‑GGGGGTACCTAAAGCCCAGCCAATCAcc                                                                                                                        <  1:608740/301‑1 (MQ=255)`  
                                                                                                          `cggagatgtgtataagagacaGGGATTCAGCGGTATGATTGCATCACACCACTTCATCCCTATAGAGTCAAGTCCTAAGGCATACCCATAAAGAGCCTCTAATGGTCTATCCTAAGGTCTATACCTAAAGATAGGCCATCCTATCAGTGTCACCTAAAGAGGGTCTTAGAGAGGGCCTATGGAGTTCCTATAGGGTCCTTTAAAATATACCATAAAAATCTGAGTGACTATCTCACAGTGTACGGACCTAAAGTTCCCACATA‑GGGGGTACCTAAAGCCCAGCCAATCACCTAAAGTCAAc                                                                                                              <  1:619676/281‑1 (MQ=255)`  
                                                                                                           `cccGACTGATGGCTAACCGAGGGATTCAGCGGTATGATTGCATCACACCACTTCATCCCTATAGAGTCAAGTCCTAAGGTATACCCATAAAGAGCCTCTAATGGTCTATCCTAAGGTCTATACCTAAAGATAGGCCATCCTATCAGTGTCACCTAAAGAGGGTCTTAGAGAGGGCCTATGGAGTTCCTATAGGGTCCTTTAAAATATACCATAAAAATCTGAGTGACTATCTCACAGTGTACGGACCTAAAGTTCCCCCATA‑GGGGGTACCTAAAGCCCAGCCAATCACCTAAAGTCAAcc                                                                                                             <  1:299216/300‑1 (MQ=255)`  
                                                                                                              `gACTGATGGCTCACCGAGGGATTCAGCGGTATGATTGCATCAAACCACTTCATCCCTATAGAGTCAAGTCATAAGGTATACCCATAAAGAGCCTCTAATGGTCTATCCTAAGGTCTATACCTAAAGATAGGCCATCCTATCAGTGTCACCTAAAGAGGGTCTTAGAGAGGGCCTATGGAGTTCCTATAGGGTCCTTTAAAATATACCATAAAAATCTGAGTGACTATCTCACAGTGTACGGACCTAAAGTTCACCCATA‑GGGGGTACCTAAAGCCCAGCCAATCACCTAAAGTCAACCTTc                                                                                                          <  1:521773/301‑1 (MQ=255)`  
                                                                                                               `aCTGATGGCTCACCTAGGGATTCAGCGGTATGATTGCATCACACCACTTCATCCCTATATTCTCAATTCCTAAGGTATACCCATAAAGAGCCTCTAATTGTCTATCCTAACGTCTATACCTAACGATAGGCCATCCTCTCAGTGTCACCTCAAGAGGGTCTTAGACAGCGCCTATGGTGTTCCTATCGGGTCCTTTACAATATACCCTAACATTCTGAGTGACTATCTCACATTTTACGTACCTACACTTCCCCCATA‑GGGGGTACCTAAAGCCCCGCCAATCACCTAAAGTCCACCTTCg                                                                                                         >  1:320878/1‑301 (MQ=255)`  
                                                                                                                `cTGATGGCTCACCGAGGGATTCAGCGGTATGATTGCATCACACCACTTCATCCCTATAGAGTCAAGTCCTAAGGTATACCCATAAAGAGCCTCTAATGGTCTATCCTAAGGTCTATACCTAAAGATAGGCCATCCTATCAGTGTCACCTAAAGAGGGTCTTAGAGAGGGCCTATGGAGTTCCTATAGGGTCCTTTAAAATATCCCATAAAAATCTGAGTAACTATCTCACAGTGTACGGACCTAACGTTCCCCCATA‑GGGGGTACCTACAGCCCAGCACATCACCTAAAGTCAACCTTCgg                                                                                                        >  1:251805/1‑301 (MQ=255)`  
                                                                                                                  `gATGGCTCACCGAGGGATTCAGCGGTATGATTGCATCACACCACTTCATCCCTATAGAGTCAAGTCCTAAGGTATACCCATAAAGAGCCTCTAATGGTCTATCCTAAGGTCTATACCTAAAGATAGGCCATCCTATCAGTGTCACCTAAAGATGGTCTTAGAGAGGGCCTATGGAGTTCCTATATGGTCCTTTAAAATATACCATAAAAATCTGAGTGACTATCTCACAGTGTACGGACCTAAAGTTCCCCCATA‑GGGGGTACTTAAAGCCCAGCCAATCACCTAAAGTCAACCTTCGGtt                                                                                                      >  1:285391/1‑301 (MQ=255)`  
                                                                                                                  `gATGGCTCACCGAGGGATTCAGCGGTATGATTGCATCACACCACTTCATCCCTATAGAGTCAAGTCCTAAGGTATACCCATAAAGAGCCTCTAATGGTCTATCCTAAGGTCTATACCTAAAGATAGGCCATCCTATCAGTGTCACCTAAAGATGGTCTTAGAGAGGGCCTATGGAGTTCCTATAGGGTCCTTTAAAATATACCATAAAAATCCGAGTGACTATCTCACAGTGTACGGACCTAAAGTTCCCCCATA‑GTGGGTACCTAAAGCCCAGCCAATCACCTAAAGTCAACCTTCGGtt                                                                                                      >  1:656535/1‑301 (MQ=255)`  
                                                                                                                                  `aTTCAGCGGTATGATTGCATCACACCACTTCATCCCTATAGAGTCAAGTCCTAAGGTATACCCATAAAGAGCCTCTAATGGTCTATCCTAAGGTCTATACCTAAAGATAGGCCATCCTATCAGTGTCACCTAAAGAGGGTCTTAGAGAGGGCCTATGGAGTTCCTATAGGGTCCTTTAAAATATACCATAAAAATCTGAGTGACTATCTCACAGTGTACGGACCTAAAGTTCACCCATA‑GGGGGTACCTAAAGCCCAGCCAATCACCTAAAGTCAACCTTAGGTTGACATTGAGGGTAccc                                                                                      >  1:118505/1‑301 (MQ=255)`  
                                                                                                                                       `gCGGTATGATTGCATCACACCACTTCATCCCTATAGAGTCAAGTCCTAAGGTATACCCATAAAGAGCCTCTAATGGTCTATCCTAAGGTCTATACCTAAAGATAGGCCATCCTATCAGTGTCACCTAAAGAGGGTCTTAGAGAGGGCCTATGGAGTTCCTATAGGGTCCTTTAAAATATACCATAAAAATCTGAGTGACTATCTCACAGTGTACGGACCTAAAGTTCCCCCATA‑GGGGGTACCTAAAGCCCACCCAATCACCTAAAGTCAACCTTCTGtctcttatacacatctccgagcc                                                                                 >  1:361388/1‑279 (MQ=255)`  
                                                                                                                                        `gggTATGATTGCATCACACCACTTCATCCCTATAGAGTCAAGTCCTAAGGTATACCCATAAAGAGCCTCTAATGGTCTATCCTAAGGTCTATACCTAAAGATAGGCCATCATATCAGTGTCACCTAAAGAGGGTCTTAGAGAGGGCCTATGGAGTTCCTATAGGGTCCTTTAAAATATACCATAAAAATCTGAGTGACTATCTCACAGTGTACGGACCTAAAGTTCCCCCATA‑GGGGGTACCTAAAGCCCAGCCAATCACCTAAAGTCAACCTTCGGTTGACCTTGAGGGTTCCATAAggg                                                                                <  1:657063/300‑1 (MQ=255)`  
                                                                                                                                        `gggTAAGATTGCATCACACCACTTCATCCCTATAGAGTCAAGTCCTAAGGTATACACATAAAGAGCCTCTAATAGTCTAACCTAAGGTCTATACCTAAAGATAGGCCATCCTATAAGTGTCACCTAAAGAGGGTCTTAGAGAGGGCCTATGGAGTTCCTATAGGGTCCTTTAAAATATACCATAAAAATCTGAGTGACTATCTCACAGTGTACGGACCTAAAGTTCCCCAATA‑GGGGGTACCTAAAGCCCAGCCAATCACCTAAAGTCAACCTTCGGTTGACCTTGAGGGTTCCCTAAggg                                                                                <  1:672848/300‑1 (MQ=255)`  
                                                                                                                                        `ggctcggagatgtgtataagggacagtCCCTATAGAGTCAAGTCCTAAGGTATACCCATAAAGAGCCTCTAATGGTCTATCCTAAGGTCTATACCTAAAGATAGGCCATCCTATCAGTGTCACCTAAAGAGGGTCTTAGAGAGGGCCTATGGAGTTCCTATAGGGTCCTTTAAAATATACCATAAAAATCTGAGTGACTATCTCACAGTGTACGGACCTAAAGTTCCCCCATA‑GGGGGTACCTAAAGCCCAGCCAATCACCTAAAGTCAACCTTCGGTTGACCTTGAGGGTTCCCTAAggg                                                                                <  1:533079/275‑1 (MQ=255)`  
                                                                                                                                        `cGGTATGATTGCATCACACCACTTCATCCCTATAGAGTCAAGTCCTAAGGTATACCCATAAAGAGCCTCTAATGGTCTATCCTAAGGTCTATACCTAAAGATAGGCCATCCTATCAGTGTCACCTAAAGAGGGTCTTAGAGAGGGCCTATGGAGTTCCTATAGGGTCCTTTAAAATATACCATAAAAATCTGAGTGACTATCTCACAGTGTACGGACCTAAAGTTCCCCCATA‑GGGGGTACCTAAAGCCCAGCCAATCACCTAAAGTCAACCTTCGGTTGACCTTGAGGGTTCCCTAAggg                                                                                <  1:482299/301‑1 (MQ=255)`  
                                                                                                                                          `gTATGATTGCATCACACCACTTCATCCCTATAGAGTCAAGTCCTAAGGTATACCCATAAAGAGACTCTAATGGTCTATCCTAAGGTCTATACCTAAAGATAGGCCATCCTATCAGTGTCACCTAAAGAGGGTCTTAGAGAGGGCCTATGGAGTTCCTATAGGGTCATTTAAAATATACCATAAAAATCTGAGTGACTATCTCACAGTGTACGGACCTAAAGTTCCCCCATA‑GGGGGTACCTAAAGCCCAGCCAATCACCTAAAGTCAACCTTCGGTTGACCTTGAGGGTTCCCTAAgggct                                                                              >  1:136674/1‑299 (MQ=255)`  
                                                                                                                                              `gATTGCATCACACCACTTCATCACTATAGAGTCAAGTCCTAAGGTATACCCATAAAGAGCCTCTAATGGTATATCCTAAGGTCTATACCTAAAGATAGGCCATCATATCAGTGTCACCTAAAGAGGGTCTTAGAGAGGGCCTATGGAGTTCCTATAGGGTCCTTTAAAATATACCATAAAAATCTGAGTGACTATCTCACAGTGTACGGACCTAAAGTTCCCCCATA‑GGGGGTACCTAAAGCCCAGCCAATCACCTAAAGTCAACCTTCGGTTGACCTTGAGGGTTCCCTAAGGGTTgggg                                                                          >  1:720274/1‑301 (MQ=255)`  
                                                                                                                                              `agtctcgtgggctcggagatgtgtataagagACAGGTCCTAAGGTATACCCATAAAGAGCCTCTAATGGTCTATCCTAAGGTCTATACCTAAAGATAGGCCATCCTATCAGTGTCACCTAAAGAGGGTCTTAGAGAGGGCCTATGGAGTTCCTATAGGGTCCTTTAAAATATACCATAAAAATCTTAGTGACTATCTCACAGTGTACGGACCTAAAGTT‑CCCCATA‑GGGGGTACCTAAAGCCCAGCCAATCACCTAAAGTCAACCTTCGGTTGACCTTGAGGGTTCCCTAAGGGTTAGGGa                                                                         <  1:523711/274‑1 (MQ=37)`  
                                                                                                                                               `aTTGCATCACACCACTTCATCCCTATAGAGTCAAGTCCTAAGGTATACCCATAAAGAGCCTCTAATGGTCTATCCTAAGGTATATACCTAAAGATAGGCCATCCTATCAGTGTCACCTAAAGAGGGTATTAGAGAGGGCCTATGGAGTTCCTATAGGGTCCTTTAAAATATACCATAAAAATCTGAGTGACTATCTCACAGTGTACGGACCTAAAGTTCCCACATA‑GGGGGTACCTAAAGCCCAGCCAATCACCTAAAGTCAACCTTCGGTTGACCTTGAGGGTTCCCTAAGGGTTGGGGa                                                                         <  1:225362/301‑1 (MQ=255)`  
                                                                                                                                                          `ccacTTCATCCCTATAGAGTCAAGTCCTAAGGTATACCCATAAAGAGCCTCTAATGGTCTATCCTAAGGTCTATACCTAAAGATAGGCCATCCTATCAGTGTCACCTAAAGAGGGTCTTAGAGAGGGCCTATGGAGTTCCTATAGGGTCCTTTAAAATATACCATAAAAATCTGAGTGACTATCTCACAGTGTACGGACCTAAAGTTCCCCCATA‑GGGGGTACCTAAAGCCCAGCCAATCACCTAAAGTCAACCTTCGGTTGACCTTGAGGGTTCCCTAAGGGTTGGGGATGACCCTTggg                                                              <  1:549270/301‑1 (MQ=255)`  
                                                                                                                                                          `ccacTTCATCCCTATAGAGTCAAGTCCTAAGGTATACCCATAAAGAGCCTCTAATGGTCTATCCTAAGGTCTATACCTAAAGATAGGCCATCCTATCAGTGTCACCTAAAGAGGGTCTTAGAGAGGGCCTATGGAGTTCCTATAGGGTCCTTTAAAATATACCATAAAAATCTGAGTGACTATCTCACAGTGTACGGACCTAAAGTTCCCCCATA‑GGGGGTACCTAAAGCCCAGCCAATCACCTAAAGTCAACCTTCGGTTGACCTTGAGGGTTCCCTAAGGGTTGGGGATGACCCTTggg                                                              <  1:293845/301‑1 (MQ=255)`  
                                                                                                                                                          `ccacTTCATCCCTATAGAGTCAAGTCCTAAGGTATACCCATAAAGAGCCTCTAATGGTCTATCCTAAGGTCTATACCTAAAGATAGGCCATCCTATCAGTGTCACCTAAAGAGGGTCTTAGAGAGGGACTATGGAGTTCCTATAGGGTCCTTTAAAATATACCATAAAAATCTGAGTGACTATCTCACAGTGTACGGACCTAAAGTT‑CCCCATA‑GGGGGTACCTAAAGCCCAGCCAATCACCTAAAGTCAACCTTCGGTTGACCTTGAGGGTTCCCTAAGGGTTGGGGATGACCCTTGGGt                                                             <  1:590016/301‑1 (MQ=255)`  
                                                                                                                                                           `cggagatgtatataagagACAGGTCCTAAGGTATACCAATAAAGAGCCTCTAATGGTCTATCCTAAGGTCTATACCTAAAGATAGGCCATCCTATCAGTGTCACCTAAAGAGGGTATTAGAGAGGGCCTATGGAGTTCCTATAGGGTCCTTTAAAATATACCATAAAAATCTGAGTGACTATCTCACAGTGTACGGACCTAAAGTTCCCCCATA‑GGGGGTACCTAAAGCCCAGCCAATCACCTAAAGTCAACCTTCGGTTGACCTTGAGGGTTCCCTAAGGGTTGGGGATGACCCTTGGGt                                                             <  1:86252/287‑1 (MQ=255)`  
                                                                                                                                                            `acTTCATCCCTAGAGAGTCAAGTCCTAAGGTATACCCAGAAAGAGCCTCTAATGGTCTATCCTAAGGGCTATACCTAAAGATAGGCCATCCTATCAGTGTCACCTAAAGAGGGTCTTAGAGAGGGCCTATGGAGTTCCTATAGGGTCCTTTAAAATATACCATAAAAATCTGAGTGACTATCTCACAGTGTACGGACCTAAAGTTCCCCCATAGGGGGGTACCTAAAGCCCAGCCAATCACCTAAAGTCAACCTTCGGTTGACCTTGAGGGTTCCCTAAGGGTTGGGGATGACCCTTGGGt                                                             <  1:420210/301‑1 (MQ=255)`  
                                                                                                                                                                  `tCCCTATAGAGTCAAGTCCTAAGGTATACCCATAAAGATCCTCTAATGGTCTATCCTAAGGTCTATACCTAAAGATAGGCCATCCTATCAGTGTCACCTAAAGAGGGTCTTAGAGAGGGCCTATTGAGTTCCTATCGATTCCGTTAAAATATATCATAAAAATCTGAGTGACTATCTCACCGTCTACGGACCTAACGTTCCACCATC‑GGGGGTACCTACAGCCCAGCCAATCACCTAAAGTCAACCTTCGGTTGACCTTGAGGGTTCCCTAAGCGTTGGGGCTgtctgttatacacatctc                                                      >  1:748979/1‑284 (MQ=37)`  
                                                                                                                                                                  `tCCCTATAGAGTCAAGTCCTAAGGTATACCCATAAAGAGCCTCTAATGGTCTATCCTAAGGTCTATACCTAAAGATAGGCCATCCTATCAGTGTCACCTAAAGAGGGTCTTAGAGAGGGCCTATGGAGTTCCTATAGGGTCCTTTAAAATATACCATAAAAATCTGAGTGACTATCTCACAGTGTACGGACCTAAAGTTCCCCCATA‑GGGGGTACCTAAAGCCCAGCCAATCACCTAAAGTCAACCTTCGGTTGACCTTGAGGGTTCCCTAAGGGTTGGGGATGACCCTTGGGTTTGTCtt                                                      >  1:58020/1‑301 (MQ=255)`  
                                                                                                                                                                    `ccTATAGAGTCAAGTCCTAAGGTATACCCATAAAGAGCCTCTAATGGTCTATCCTAAGGTCTATACCTAAAGATAGGCCATCCTATCAGTGTCACCTAAAGAGGGTCTTAGAGAGGGCCTATGGAGTTCCTATAGGGTCCTTTAAAATATACCATAAAAATCTGAGTTACTATCTCACAGTGTACGGACCTAAAGTTCCCCCATA‑GGGGGTACCTAAAGCCCAGCCAATCACCTAAAGTCAACCTTCGGTTGACCTTGAGGGTTCCCTAAGGGTTGGGGATGACCCTTgggctgtctctta                                                    >  1:818753/1‑291 (MQ=255)`  
                                                                                                                                                                     `cTATAGGGTCAAGTCCTAAGGTATACCCATAAAGAGCCTCTAATGGTCTATCCTAAGGTCTATACCTAAAGATAGGCCATCCTATCAGTGTCACCTAAAGAGGGTCTTAGAGAGGGCCTATGGAGTTCCTATAGGGTCCTTTAAAATATACCATAAAAATCTGAGTGACTATCTCACAGTGTACGGACCTAAAGTT‑CCCCATA‑GGGGGTACCTAAAGCCCAGCCAATCACCTAAAGTCAACCTTCGGTTGACCTTGAGGGTTCCATAAGGGTTGGGGATGACCCTTGGGTTTGTCTTTggg                                                  <  1:147735/301‑1 (MQ=255)`  
                                                                                                                                                                     `cTATAGAGTCAAGTCCTAAGGTATACCCATAAAGAGCCTCTAATGGTCTATCCTAAGGTCTATACCTAAAGATAGGCCATCCTATCAGTGTCACCTAAAGAGGGTCTTAGAGAGGGCCTATGGAGTTCCTATAGGGTCCTTTAAAATATACCATAAAAATCTGAGTGACTATCTCACAGTGTACGGACCTAAAGTTCCCCCATA‑GGGGGTACCTAAAGCCCAGCCAATCACCTAAAGTCAACCTTCGGTTGACCTTGAGGGTTCCCTAAGGGTTGGGGactgtctcttatacacatctccg                                                   >  1:241292/1‑279 (MQ=255)`  
                                                                                                                                                                     `cTATAGAGTCAAGTCCTAAGGTATACCCATAAAGAGCCTCTAATGGTCTATCCTAAGGTATATACCTAAAGATAGGCCATCCTATCAGTGTCACCTAAAGAGGGTCTTAGAGAGGGCCTATGGAGTTCCTATAGGGTCCTTTAAAATATACCATAAAAATCTGAGTGACTATCTCACAGTGTACGGACCTAAAGTT‑CCCCATA‑GGGGGTACCTAAAGCCCAGCCAATCCCCTAAAGTCAACCTTCGGTTGCCCTTGAGGGTTCCCTAAGGGTTGGGGactgtctcttatacacctctccga                                                  >  1:519007/1‑278 (MQ=255)`  
                                                                                                                                                                     `cTATAGAGTCAAGTCCTAAGGTATACCAATAAAGAGCCTCTAATGGTCTATCCTAAGGTCTATACCTAAAGATAGGCCATCCTATCAGTGTCACCTAAAGGGGGTCTTAGAGAGGGCCTATGGAGTTCCTATAGGGTCCTTTAAAATATACCATAAAAATCTGAGTGACTATCTCACAGTGTACGGACCTAAAGTTCCACAATA‑GGGGGTACCTAAAGCCCAGCAAATCACCTAAAGTCAACCTTCGGTTGACCTTGAGGGTTCCCTAAGGGTTGGGGATGACCCTTgggctgtctcttat                                                   >  1:95986/1‑290 (MQ=255)`  
                                                                                                                                                                     `agatgtgtataagagacagggTATACCCATAAAGAGCCTCTAATGGTCTATCCTAAGGTCTATACCTAAAGATAGGCCATCCTATCAGTGTCACCTAAAGAGGGTCTTAGAGAGGGCCTATGGAGTTCCTATAGGGTCCTTTAAAATATACCATAAAAATCTGAGTGACTATCTCACAGTGTACTGACCTAAAGTT‑CCCCATA‑GGTGGTACCTAAAGCCCAGCCAATCACCTAAAGTCAACCTTCGGTTGACCTTGAGGGTTCCCTAAGGGTTGGGGATGACCCTTGGGTTTGTCTTTggg                                                  <  1:456956/282‑1 (MQ=255)`  
                                                                                                                                                                      `gtaggagatgtgtataagagacagcccATAAAGAGCCTCTAATGGTCTATCCTAAGGTCTATACCTAAAGATAGGCCATCCTATCAGTGTCACCTAAAGAGGGTCTTAGAGAGGGCCTATGGAGTTCCTATAGGGTCCTTTAAAATATACCATAAAAAGCTGAGTGACTATCTCACAGTGTACGGACCTAAAGTTCCCCCATA‑GGGGGTACCTAAAGCCCAGCCAATCAACTAAAGTCAACCTTCGGTTGACCTTGAGGGTTCCCTAAGGGTTTGGGATGACCCTTGGGTTTGTCTTTggg                                                  <  1:217328/277‑1 (MQ=37)`  
                                                                                                                                                                      `ctcggagatgtgtataagagacagcccATAAAGAGCCTCTAATGGTCTATCCTAAGGTCTATACCTAAAGATAGGCCATCCTATCAGTGTCACCTAAAGAGGGTCTTAGAGAGGGCCTATGGAGTTCCTATAGGGTCCTTTAAAATATACCATAAAAATCTGAGTGACTATCTCACAGTGTACGGACCTAAAGTTCCCCCATA‑GGGGGTACCTAAAGCCAAGCCAATCACCTAAAGTCAACCTTCGGTTGACCTTGAGGGTTCCCTAAGGGTTGGGGATGACCCTTGGGTTTGTCTTTggg                                                  <  1:194871/277‑1 (MQ=255)`  
                                                                                                                                                                            `gTCAAGTCCTAAGGTATACCCATAAAGAGCCTCTAATGGTATATCCTAAGGTCTATACCTAAAGATAGGCCATCCTATCAGTGTCACCTAAAGAGGGTCTTAGAGAGGGCCTATGGAGTTCCTATAGGGTCCTTTAAAATATACCATAAAAATCTGAGTGACTATCTAACAGTGTACGGACCTAAAGTTCCCCCATA‑GGGGGTACCTAAAGCCCAGTCAATCACCTAAAGTCAACCTTCGGTTGACCTTGAGGGTTCCCTAAGGGTTGGGGATGACGCTTGGGTTTGTCTTTGGGTGTTAc                                            <  1:201232/301‑1 (MQ=255)`  
                                                                                                                                                                             `tCAAGTCCTAAGGTATACCCATAAAGAGCCTCTAATGGTCTATCCTAAGGTCTATACATAAAGATAGGCCATCCTATCAGTGTCACCTAAAGAGGGTCTTATAGAGGGCCTATGGAGTTCCTATAGGGTCCTTTAAAATATACCATAAAAATCTGAGTGACTATCTCACAGTGTACGGACCTAAAGTTCCCCCATA‑GGGGGTACCTAAAGCCCAGCCAATCACCTAAAGTCAACCTTCGGTTGACCTTGAGGGTTCCCTAAGGGTTGGGGATGACCCTTGGGTTTGTCTTTGGGTGTTAcc                                           <  1:154740/301‑1 (MQ=255)`  
                                                                                                                                                                             `gctcggagatgtgtataagagacaggagCCTCTAATTGTCTATCCTAAGGTCTATACCTAAAGATAGGCCATCCTATCAGTGTCACCTAAAGAGGGTCTTAGAGAGGGCCTATGGAGTTCCTATAGGGTCCTTTAAAATATACCATAAAAATCTGAGTGACTATCTCACAGTGTACGGACCTAAAGTTCCCCCATA‑GGGGGTACCTAAAGCCCAGCCAATCACCTAAAGTCAACCTTCGGTTGACCTTGAGGGTTCCCTAAGGGTTGGGGATGACCCTTGGGTTTGTCTTTGGGTGTTAcc                                           <  1:704089/276‑1 (MQ=255)`  
                                                                                                                                                                              `cAAGTCCTAATGTATACCCATAAAGAGCCTCTAATGGTCTATCCTAAGGTCTATACCTAAAGATAGGCCATCCTATCAGTGTCACCTAAAGAGGGTCTTAGAGAGGGCCTATGGAGTTCCTATAGGGTCCTTTAAAATATACCATAAAAATCTGAGTGACTATCTCACAGTGTACGGACCTAAAGTTCCCCCATA‑GGGGGTACCTAAAGCCCAGCCAATCACCTAAAGTCAACCTTCGGTTGACCTTGAGGGTTCCCTAAGGGTTGGGGATGACCCTTGGGTTTGTCTTTGGGTGTTACCt                                          <  1:191326/301‑1 (MQ=255)`  
                                                                                                                                                                              `cAAGTCCTAAGGTATACCCATAAAGAGCCTCTAATGGTCTATCCTAAGGTCTATACCTAAAGATAGGCCATCCTATCAGGGTCACCTAAAGAGGGTCTTAGAGAGGGCCTATGGAGTTCCTATAGGGTCCTTTAAAATATACCATAAAAATCTGAGTGACTATCTCACAGTGTACGGACCTAAAGTTCCCCCATA‑GGGGGTACCTAAAGCCCAGCCAATCACCTAAAGTCAACCTTCTGTTGACCTTGAGGGTTCCCTAATGGTTGGTGATGACCCTTGGGTTTGTCTTTGGGTGTTACCt                                          <  1:740123/301‑1 (MQ=255)`  
                                                                                                                                                                              `aaaGTCCTAAGGTATACCCATAAAGAGCCTCTAATGGTCTATCCTAAGGTCTATACCTAAAGATAGGCCATCCTATCAGTGTCACCTAAAGAGGGTCTTAGAGAGGGCCTATGGAGTTCCTATAGGGTCCTTTAAAATATACCATAAAAATCTGAGTGACTATCTCACAGTGTACGGACCTAAAGTTCCCCCATA‑GGGGGTACCTAAAGCCCAGCCAATCACCTAAAGTCAACCTTCGGTTGACCTTGAGGGTTCCCTAAGGGTTGGGGATGACCCTTGGGTTTGTCTTTGGGTGTTACCt                                          <  1:379516/300‑1 (MQ=255)`  
                                                                                                                                                                                `aGTCCTAAGGTATACCCATTAAGCGCCTCTAATGGTCTATCCTAAGGTCTATACTTAAAGATAGGCCATCCTATCAGTGTCACCTAAAGAGGGTCTTAGAGAGGGCCTATGGAGTTCCTATAGGGTCCTTTAAAATATACCATAAAAATCTGAGTGACTATCTCACAGTGTACGGACCTGAAGTTCTCCCATA‑GTGGGTACCTAAAGCCCAGCCAATCACCTAAAGTCAACCTTCGGTTGACCTTGAGGGTTCCCTAAGGGTTGTGGATGACCCTTTGGTTTGTCTTTGGGTGTTACCTTg                                        <  1:252856/301‑1 (MQ=255)`  
                                                                                                                                                                                  `tataagagacagcccATAAAGAGCCTCTAATGGTCTATCCTAAGGTCTATACCTAAAGATAGGCCATCCTATCAGTGTCACCTAAAGAGGGTCTTAGAGAGGGCCTATGGAGTTCCTATAGGGTCCTTTAAAATATACCATAAAAATCTGAGTGACTATCTCACAGTGTACGGACCTAAAGTTCCCCCATA‑GGGGGTACCTAAAGCCCAGCCAATCACCTAAAGTCAACCTTCGGTTGACCTTGAGGGTTCCCTAAGGGTTGGGGATGACCCTTGGGTTTGTCTTTGGGTGTTACCTTGAg                                      <  1:14175/289‑1 (MQ=255)`  
                                                                                                                                                                                  `tCCTAAGGTATACCCATAAAGAGCCTCTAATGGTCTATCCTAAGGTCTATACCTAAAGATAGGCCATCCTATCAGTGTCACCTAAAGAGGGTCTTAGAGAGGGCCTATGGAGTTCCTATAGGGTCCTTTAAAATATACCATAAAAATCTGAGTGACTATCTCACAGTGTACGGACCTAAAGTTCCCCCATA‑GGGGGTACCTAAAGCCAAGCCAATCACCTAAAGTCAACCTTCGGTTGACCTTGAGGGTTCCCTAAGGGTTGGGGATGACCCTTGGgtctgtctcttatacacatctcctc                                      >  1:501493/1‑284 (MQ=255)`  
                                                                                                                                                                                    `cTAAGGTATACCCATAAAGAGCCTCTAATGGTCTATCCTAAGGTCTATACCTAAAGATAGGCCATCCTATCAGTGTCACCTAAAGAGGGTCTTAGAGAGTGCCTATGGAGTTCCTATAGGGTCCTTTAAAATATACCATAAAAATCTGAGTGACTATCTCACAGTGTACGGACCTAAAGTTCCCCCATA‑GGTGGTACCTAAAGCCCAGCCAATCACCTAAAGTCAACCTTCGGTTGACCTTGAGGGTTCCCTAAGGGTTGGGGATGACCCTTGGGTTTGTCTTTGGGTGTTACCTTGAgtg                                    <  1:614278/301‑1 (MQ=255)`  
                                                                                                                                                                                      `aaGGTATACCCATAAAGATCCTCTAATGGTCTATCCTAAGGTCTATACCTAAAGATAGGCCATCCTATCAGTGTCACCTAAAGAGGGTCTTAGAGAGGGCCTATGGAGTTCCTATAGGGTCCTTTAAAATATACCATAAAAATCTGAGTGACTATCTCACAGTGTACGGACCTAAAGTT‑CCCCATA‑GGGGGTACCTAAAGCCCAGCCAATCACCTAAAGTCAACCTTCGGTTGACCTTGATGGTTCCCTAAGGGTTGTGGATGACCCTTGGGTTTGTCTTTGGGTGTTACCTTGAGTGtct                                 >  1:101875/1‑301 (MQ=255)`  
                                                                                                                                                                                      `aaGGTATACCCATAAAGAGCCTCTAATGGTCTATCCTAAGGTCTATACCTAAAGATAGGCCATCCTATCAGTGTCACCTAAAGAGGGTCTTAGAGAGGGCCTATGGAGTTCCTATAGGGTCCTTTAAAATATACCATAAAAATCTGAGTGACTATCTCACAGTGTACGGACCTAAAGTTCCCCCATA‑GGGGGTACCTAAAGCCCAGCCAATCACCTAAAGTCAACCTTCGGTTGACCTTGAGGGTTCCCTAAGGGTTGGGGATGACCCTTGGGTTTGTCTTTGGGTGTTACCTTGAGTGtc                                  <  1:475400/301‑1 (MQ=255)`  
                                                                                                                                                                                        `ggTATACCCATAAAGAGCCTCTAATGGTCTATTCTAAGGTCTATACCTAAAGATAGGCCATCCTATCAGTGTCACCTAAAGAGGGTCTTAGAGAGGGCCTATGGAGTTCCTATAGGGTCCTTTAAAATATACCATAAAAATCTGAGTGACTATCTCACAGTGTACGGACCTAAAGTTCCCCCATA‑GGGGGTACCTAAAGCCCAGCCAATCACCTAAAGTCAACCTTCGGTTGACCTTGAGGGTTCCCTAAGGGTTGGGGATGACCCTTGGGTTTGTCTTTGGGTGTTACCTTGAGTGtctc                                <  1:497509/301‑1 (MQ=255)`  
                                                                                                                                                                                        `ggTATACCCATAAAGAGCCTCTAATGGTCTATCATAAGGTCTATACCTAAAGATAGGACATCCTATCAGTGTCACCTAAAGAGGGTCTTAGAAAGGGCCTATGGAGTTCCTATAGGGTCCTTTAAAATATACCATAAAAATCTGAGTGACTATCTCACAGTGTACGGACCTAAAGTT‑CCCCATA‑GGGGGTACCTAAAGCCCAGCCAATCACCTAAAGTCAACCTTCGGTTGACCTTGAGGGTTCCCTAAGGGTTGGGGATGACCCTTGGGTTTGTCTTTGGGTGTTACCTTGAgtctgtct                               >  1:516080/1‑301 (MQ=255)`  
                                                                                                                                                                                          `tataCCCATAAAGAGCCTCTAATGGTCTATCCTAAGGTCTATACCTAAAGATAGGCCATCCTATCAGTGTCACCTAAAGAGGGTCTTAGAGAGGGCCTATGGAGTTCCTATAGGGTCCTTTAAAATATACCATAAAAATCTGAGTGACTATCTCACAGTGTACGGACCTAAAGTTCCCCCATA‑GGGGGTACCTAAAGCCCAGCCAATCACCTAAAGTCAACCTTCGGTTGACCTTGAGGGTTCCCTAAGGGTTGGGGATGACCCTTGGGTTTGTCTTTGGGTGTTACCTTGAGTGtctctc                              <  1:438111/301‑1 (MQ=255)`  
                                                                                                                                                                                           `ataCCCATAAAGAGCCTCTAATGGTCTATCCTAAGGTCTATACCTAAAGATAGGCCATCCTATCAGTGTCACCTAAAGAGGGTCTTAGAGAGGGCCTATGGAGTTCCTATAGGGTCCTTTAAAATATACCATAAAAATCTGAGTGACTATCTCACAGTGTACGGACCTAAAGTT‑CCCCATA‑GGGTGTACCTAAAGCCCAGCCAATCACCTAAAGTCAACCTTCGGTTGACCTTGAGGGTTCCCTAAGGGTTGGGGATGACCCTTGGGTTTGTCTTTGGGTGTTACCTTGAGTGTCTCTCtg                            <  1:634554/301‑1 (MQ=255)`  
                                                                                                                                                                                            `tgtgtataagagacagctAATGGTCTATCCTAAGGTCTATACCTAAAGATAGGCCATCCTATCAGTGTCACCTAAAGAGGGTCTTAGAGAGGGCCTATGGAGTTCCTATATGGTCCTTTAAAATATACCATAAAAATCTGAGTGACTATCTCACAGTGTACGGACCTAAAGTTCCCCCATA‑GGGGGTACCTAAAGCCCAGCCAATCACCTAAAGTCAACCTTCGGTTGACCTTGAGGGTTCCCTAAGGGTTGGGGATGACCCTTGGGTTTGTCTTTGGGTGTTACCTTGAGTGTCTCTCtg                            <  1:640143/285‑1 (MQ=255)`  
                                                                                                                                                                                            `taCCCATAAAGAGCCTCTAATGGTCTATCCTAAGGTCTATACCTAAAGATAGGCCATCCTATCAGTGTCACCTAAAGAGGGTCTTAGAGATGGCCTATGGAGTTCCTATAGGGTCCTTTAAAATATACCATAAAAATCTGAGTGACTATCTCACAGTGTACGGACCTAAAGTTCCCCCTTA‑GGGGGTACCTAAAGCCCAGCCAATCACCTAAAGTCAACCTTCGGTTGACCTTGAGGGTTCCCTAAGGGTTGGGGATGACCCTTTGGTTTGTCTTTGGGTGTTACCTTGAGTGTCTATCtg                            <  1:534047/301‑1 (MQ=255)`  
                                                                                                                                                                                            `ggctcggagatgtgtataagagacagaTCCTAAGGTCTATACCTAAAGATAGGCAATCCTATCAGTGTCACCTAAAGATGGTCTTAGAGAGGGCCTATGTAGTTCCTATAGGGTCCTTTAAAATATACCATAAAAATCTGAGTGACTATCTCACAGTGTACGGACCTAAAGTTCCCCCATA‑GGGGGTACCTAAAGCCCAGCCAATCACCTAAAGTCAACCTTCGGTTGACCTTGAGGGTTCCCTAAGGGTTGTGGATGACCCTTGGGTTTGTCTTTGGGTGTTACCTTGAGTGTCTCTCtg                            <  1:802153/275‑1 (MQ=21)`  
                                                                                                                                                                                            `aaCCCATAAAGAGCCTCTAATGGTCTATCCTAAGGTCTATACCTAAAGATAGGCCATCCTATCAGTGTCACCTAAAGAGGGTCTTAGAGAGGGCCTATGGAGTTCCTATAGGGTACTTTAAAATATACCATAAAAATCTGAGTGACTATCTCACAGTGTACGGACCTAAAATTCCCCCATA‑GGGGGTACCTAAAGCCCAGCCAATCACCTAAAGTCAACCTTCGGTTGACCTTGAGGGTTCCATAAGGGTTGGGGATGACCCTTGGGTTTGTCTTTGGGTGTTACCTTGAGTGTCTCTCtg                            <  1:124265/300‑1 (MQ=255)`  
                                                                                                                                                                                              `cccATAAAGAGCCTCTAATGGTCTATCCTAAGGTCTATACCTAAAGATAGGCCATCCTATCAGTGTCACCTAAAGAGGGTCTTAGAGAGGGCCTATGGAGTTCCTATAGGGTCCTTTAAAATATACCATAAAAATCTGAGTGACTATCTCACAGTGTACGGACCTAAAGTTCCCCCATA‑GGTGGTACCTAAAGCCCAGCCAATCACCTAAAGTCAACCTTCGGTTGACCTTGAGGGTTCCCTAAGGGTTGGGGATGACCCTTGGGTTTGTCTTTGGGTGTTACCTTGAGTGTCTCTCtgtg                          <  1:806293/301‑1 (MQ=255)`  
                                                                                                                                                                                              `cccATAAAGAGCCTCTAATGGTCTATCCTAAGGTCTATACCTAAAGATAGGCCATCCTATCAGTGTCACCTAAAGAGGGTCTTAGAGAGGGCCTATGGAGTTCCTATAGGGTCCTTTAAAATATACCATAAAAATCTGAGTGACTATCTCACAGTGTACGGACCTAAAGTTCCCCCATA‑GGGGGTACCTAAAGCCCAGCCAATCACCTAAAGTCAACCTTCGGTTGACCTTGAGGGTTCCCTAAGGGTTGGGGATGACCCTTGGGTTTGTCTTTGGGCGTTACCTTGAGTGtatctcctgt                          >  1:426966/1‑297 (MQ=255)`  
                                                                                                                                                                                                          `ctctAATTGTCTATCCTAAGGTCTATACCTAAAGATAGGCCATCCTATCAGTGTCACCTAAAGAGGGTCTTAGAGAGGGCCTATGGAGTTCCTATAGGGTCCTTTAAAATATACCATAAAAATCTGAGTGACTATCTCACAGTGTACGGACCTAAAGTTCCCCCATA‑GGGGGTACCTAAAGCCCAGCTAATCACCTAAAGTCAACCTTCGGTTGACCTTGAGGGTTCCCTAAGGGTTGGGGATGACCCTTGGGTTTGTCTTTTGGTGTTACCTTGAgtgctgtctcttatacacatctcct              >  1:288146/1‑279 (MQ=25)`  
                                                                                                                                                                                                                      `aTCCTAAGGTCTATACCTAAAGATAGGCCATCCTATCAGTGTCACCTAAAGATGGTCTTAGAGAGGGCCTATGGATTTCCTATAGGGTCCTTTAAAATATACCATAAAAATCTGAGTGACTATCTCACATTGTACGGACCTAAAGTTCCCCCATA‑GGGGGTACCTAAAGCCCAGCCAATCACCTAAAGTCAACCTTCGGTTGACCTTGCGGGTTCCCTAAGGGTTGGGGATTACCCTTGGGTTTGTCTTTGGTTGTTACCTTGAGTGTCTCTCtgctgtctcttatacacatctccgagcc  >  1:46508/1‑275 (MQ=21)`  
                                                                                                                                                                                                                      `aTCCTAAGGTCTATACCTAAAGATAGGCCATCCTATCAGTGTCACATAAAGAGGGTCTTAGAGAGGGCCTATGGAGTTCCTGTAGGGTCCTTAAAAATATACCATAAAAATCTGAGTGACTATCTCACAGTGTACGGACCTACAGTTCCCCCATA‑GGGGGTACCTAAAGCCCAGCCAATCACCTAAAGTCAACCTTCGGTTGACCTTGAGGGTTCCCTAAGGGTTGGGGATGACCCTTGGGTTTGTCTTTGGGTGTTACCTTGAGTGTCTCTCtgctgtctcttatacacatctccgagcc  >  1:301512/1‑275 (MQ=21)`  

                                                                                                                                                                                                                                                                                                            `|`                                                                                                                                                                                                                         
`TGTGGTACTTTGTGCTGCCCTTGGGTACGCATCTCTTACTGGAGACCTCAGTTCACTGGAGTCTGTCGTTTGCTCTATACTCACTTGTAGCGATTAGGGTCTTCCTGACCGACTGATGGCTCACCGAGGGATTCAGCGGTATGATTGCATCACACCACTTCATCCCTATAGAGTCAAGTCCTAAGGTATACCCATAAAGAGCCTCTAATGGTCTATCCTAAGGTCTATACCTAAAGATAGGCCATCCTATCAGTGTCACCTAAAGAGGGTCTTAGAGAGGGCCTATGGAGTTCCTATAGGGTCCTTTAAAATATACCATAAAAATCTGAGTGACTATCTCACAGTGTACGGACCTAAAGTTCCCCCATA‑GGGGGTACCTAAAGCCCAGCCAATCACCTAAAGTCAACCTTCGGTTGACCTTGAGGGTTCCCTAAGGGTTGGGGATGACCCTTGGGTTTGTCTTTGGGTGTTACCTTGAGTGTCTCTCTGTG‑‑‑‑‑‑‑‑‑‑‑‑‑‑‑‑‑‑‑TCCCT  >  NC_001604.1/39442‑39937`

  

Alignment Legend

Aligned base mismatch/match (shaded by quality score): `ATCG/ATCG` `< 3 ≤` `ATCG/ATCG` `< 12 ≤` `ATCG/ATCG` `< 27 ≤` `ATCG/ATCG` `< 37 ≤` `ATCG/ATCG` `< 38 ≤` `ATCG/ATCG`

Unaligned base: `atcg`    Masked matching base: `atcg`    Alignment gap: `‑`    Deleted base: `‑`
